# Genetic variants in guanylyl cyclase and phoshodiesterase affecting coronary disease risk

**DOI:** 10.1186/2050-6511-14-S1-O6

**Published:** 2013-08-29

**Authors:** Heribert Schunkert

**Affiliations:** 1 Deutsches Herzzentrum München, Technische Universität München, Lazarettstraße 36, Germany

## 

Impaired guanylyl cyclase activity (sGC) may be considered as a cardio-vascular risk factor. In order to study such association with genetic tools, we investigated the role of the GUCY 1A3 locus in genomewide association studies as well as families with premature coronary artery disease. We discovered genomewide significance for association between rs7692387 with coronary artery disease / myocardial infarction in the CARDIoGRAMplusC4D study. Moreover, we discovered mutations in the GUCY 1A3 gene in families with premature coronary disease. Functional studies revealed that impaired guanylyl cyclase activity was the most likely functional link between the genetic variations and the increased risk of coronary disease.

